# Irregular word reading as a marker of cognitive and semantic decline in Alzheimer’s disease rather than an estimate of premorbid intellectual abilities

**DOI:** 10.21203/rs.3.rs-3381469/v1

**Published:** 2023-09-29

**Authors:** Anna Marier, Mahsa Dadar, Florence Bouhali, Maxime Montembeault

**Affiliations:** Department of Psychology, Université de Montréal, C.P. 6128, succursale Centre-Ville, Montréal, QC, Canada, H3C 3J7; Douglas Research Centre & Department of Psychiatry, McGill University, 6875 Boulevard LaSalle, Montréal, QC, Canada, H4H 1R3; Aix-Marseille University; Douglas Research Centre & Department of Psychiatry, McGill University, 6875 Boulevard LaSalle, Montréal, QC, Canada, H4H 1R3

**Keywords:** Alzheimer’s dementia, premorbid intelligence, verbal intelligence, irregular word, exception word, reading, semantic, neuropsychology, mild cognitive impairment, deformation-based morphometry

## Abstract

**Background:**

Irregular word reading has been used to estimate premorbid intelligence in Alzheimer’s disease (AD) dementia. However, reading models highlight the core influence of semantic abilities on irregular word reading, which shows early decline in AD. The general aim of this study is to determine whether irregular word reading is a valid estimate of premorbid intelligence, or a marker of cognitive and semantic decline in AD.

**Method:**

681 healthy controls (HC), 104 subjective cognitive decline, 290 early and 589 late mild cognitive impairment (EMCI, LMCI) and 348 AD participants from the Alzheimer’s Disease Neuroimaging Initiative were included. Irregular word reading was assessed with the American National Adult Reading Test (AmNART). Multiple linear regressions were conducted predicting AmNART score using diagnostic category, general cognitive impairment and semantic tests. A generalized logistic mixed-effects model predicted correct reading using extracted psycholinguistic characteristics of each AmNART words. Deformation-based morphometry was used to assess the relationship between AmNART scores and voxel-wise brain volumes, as well as with the volume of a region of interest placed in the left anterior temporal lobe (ATL).

**Results:**

EMCI, LMCI and AD patients made significantly more errors in reading irregular words compared to HC, and AD patients made more errors than all other groups. Across the AD continuum, as well as within each diagnostic group, irregular word reading was significantly correlated to measures of general cognitive impairment / dementia severity. Neuropsychological tests of lexicosemantics were moderately correlated to irregular word reading whilst executive functioning and episodic memory were respectively weakly and not correlated. Age of acquisition, a primarily semantic variable, had a strong effect on irregular word reading accuracy whilst none of the phonological variables significantly contributed. Neuroimaging analyses pointed to bilateral hippocampal and left ATL volume loss as the main contributors to decreased irregular word reading performances.

**Conclusions:**

Irregular word reading performances decline throughout the AD continuum, and therefore, premorbid intelligence estimates based on the AmNART should not be considered accurate in MCI or AD. Results are consistent with the theory of irregular word reading impairments as an indicator of disease severity and semantic decline.

## Background

Alzheimer’s disease (AD) is the most common cause of dementia (Alzheimer’s Association, 2023). It is characterized by the insidious accumulation of beta-amyloid and tau proteins ensuing damage to neurons and accompanied with progressive cognitive and behavioral changes. The AD continuum is characterized by three phases, (a) preclinical, (b) mild cognitive impairment (MCI) and (c) AD dementia. One condition which has received increasing attention as an indicator of preclinical AD is subjective cognitive decline (SCD), described as the perception (by oneself or a close contact) of worsening of one’s mental abilities, despite seemingly unimpaired performance on objective tests (Jessen et al., 2014). SCD has been associated with increased risks of future objective cognitive decline (Mitchell et al., 2014), as well as increased likelihood of biomarker abnormalities consistent with AD pathology (Visser et al., 2009). In the intermediate stage between SCD and AD dementia, MCI patients present objective impairment in one or more cognitive domains (Albert et al., 2011), but their cognitive changes are mild enough that they require minimal aid or assistance, retaining independence of function in their daily life. On the other hand, AD dementia is associated with more significant cognitive impairments in at least two cognitive domains, which in this case interferes with independence and activities of daily living (Mckhann et al., 2011). The typical amnestic AD dementia most prominently affects learning of new information (episodic memory), but deficits can also be observed in language, visuospatial or executive functions, and through behavioral abnormalities or personality changes. Measuring cognitive decline is therefore central in assessing individuals on the AD continuum. To establish cognitive decline, clinicians will often rely on self and relative-reported changes, as well as comparisons to demographically-adjusted norms of cognitive performance in healthy individuals. Another method is to compare current abilities to an estimate of one’s baseline abilities before they were affected by the disease, often referred to as premorbid abilities.

Historically and across many countries, one of the ways to estimate premorbid abilities in patients is the administration of irregular word reading tests (Nelson, 1982; Blair and Spreen, 1989; Schmand et al., 1991; Grober and Sliwinski, 1991; Beardsall and Huppert, 1994; Del Ser et al., 1997; Vaskinn et Sundet, 2001; Ginsberg et al., 2003; Mackinnon and Mulligan 2005; Matsuoka et al., 2006; Tallberg et al., 2006; Rolstad et al., 2008; Starkey and Halliday, 2011; Alves et al., 2012; Yi et al., 2017). It relies on the assumptions that (a) reading abilities reached by a normal adult is related to their general intelligence and (b) once reading becomes a highly practiced and overlearned skill, it can be maintained at a high level despite deteriorations in other areas of intellectual functioning (Nelson and McKenna, 1975). In 1978, Nelson and O’Connell introduced the first irregular word reading test, the New (later changed to “National”) Adult Reading Test (NART). The logic behind the use of irregular word reading, as opposed to regular word reading in estimating premorbid intelligence, is that irregular word reading relies on familiarity to specific words with exceptional spelling. For example, “pint” can only be read correctly by a person who know of the word and recognises it. Its pronunciation indeed cannot be guessed through the application of common rules of grapheme-phoneme correspondence, as that would only result in reading it like “mint”. Therefore, the accurate reading of less frequent irregular words would indicate a larger premorbid vocabulary, which would be related to a high premorbid intellectual quotient (IQ). This assumption was verified on many occasions in healthy adults, most recently when the NART was standardized against the Weschler Adult Intelligence Scale IV, both tests correlating with a correlation coefficient of *r*= .69 (Bright et al., 2018).

While this assumption was mainly tested in healthy adults, the situation might be different in neurodegenerative disorders. In 1996, Taylor and colleagues pointed out that if estimates of premorbid IQ in patients with neurodegenerative disorders are to be considered valid and accurate, they should (a) not significantly change as disease progresses in severity and (b) not differ significantly from those of demographically matched control subjects (i.e., cognitively unimpaired older adults). These assumptions are consistent with the fact that crystallized intelligence remains relatively stable across the lifespan. In line with these criteria, many cross-sectional studies support the use of NART-like tests in estimating premorbid IQ of AD patients (Nebes et al., 1984; O’Carroll and Gilleard, 1986; Cummings et al., 1986; Hart et al., 1986; Crawford et al., 1988; Schlosser and Ivison, 1989; Sharpe and O’Carroll, 1991; Raymer and Berndt, 1996; Johnstone et al., 1996; Maddrey et al., 1996; Smith, 1997; Paolo et al., 1997; Law and O’Carroll, 1998; Bright et al., 2002; Luzzatti et al., 2003; McGurn et al., 2004; Matsuoka et al., 2006; Alves et al., 2013; Yi et al., 2017). According to those studies, their respective NART-like tests are (at least sufficiently) dementia-insensitive to provide a useful measure of premorbid IQ. This assumption results from the fact that they have not observed significant differences on NART-like test scores between demographically-matched healthy controls (HC) and AD participants, despite those groups differing significantly on regular IQ tests. This absence of group differences would indicate, at least in theory, that the NART-like score of those AD participants has not declined significantly since before they developed neurodegenerative symptoms, that the difference between the NART estimated IQ and the current IQ would serve to quantify the AD patient’s true cognitive decline. In addition to this cross-sectional support, one longitudinal study found that irregular word reading scores remained stable over a period of 2 years in mild AD patients (Rolstad et al., 2008). In conflict with these conclusions however, many cross-sectional studies have observed significant differences on NART-like test scores between demographically matched HC and AD participants, thus giving support to the theory that irregular word reading might be affected in AD dementia and that this widely used test does not give an accurate estimate of premorbid intelligence in this population (Rapcsak et al., 1989; Patterson et al., 1994; O’Carroll et al.,1995; Storandt et al., 1995; Hughes et al., 1997; Conway et O’Carroll, 1997; Glosser et al., 1999; Taylor, 2000; Pestell et al., 2000; Weekes, 2000; Colombo et al., 2000; Noble et al, 2000; Colombo et al., 2004; McFarlane et al., 2006). Even more importantly, several longitudinal studies also offer support to this theory in that they observed a significant decline in NART-like performance in AD participants over time (Fromm et al., 1991; Paque and Warrington, 1995; Taylor et al., 1996; Strain et al., 1998; Cockburn et al., 2000; Pavlik et al., 2006; Grober et al., 2008; Lowe Rogers, 2011; Weinborn et al.,2018).

These conflicting lines of research could be the result of different factors. A first problem with many of the aforementioned studies is that they have been conducted in the 90s and early 2000s, when concepts like SCD and MCI didn’t exist. The same can be said for biomarkers and AD dementia criteria which were not as well developed at the time (McKhann et al., 2011). In those older studies, it is possible that SCD or MCI participants were classified as normal controls or that other types of dementias were diagnosed as AD dementia. Of note, SCD patients are absent from all the aforementioned studies whilst only three included MCI participants (that is, Alves et al., 2013; Yi et al., 2017; Weinborn et al., 2018). Previous studies were also conducted using a relatively low sample size, most often with less than 50 AD participants. This brings particular concern towards the studies in support of the accuracy of irregular word reading premorbid IQ estimates in AD dementia, because in some, nonsignificant differences suggested that a larger sample size would reveal statistical and clinical significance in control-AD comparisons (Nebes et al., 1984; Maddrey et al., 1996; Paolo et al., 1997). Nonetheless, when focusing on studies with larger samples sizes and/or longitudinal studies (vs. cross-sectional studies) the evidence seems against the use of irregular word reading as a marker of premorbid IQ in AD dementia. It is also notable that even in studies supporting their use, NART-like tests were often found to only be accurate at certain, earlier stages of the AD continuum, whilst becoming inaccurate in more severe stages. The stage at which inaccuracies appear varies from study to study, ranging from MCI to moderately severe AD.

Alternatively to the theory that irregular word reading is a measure of premorbid intelligence in AD dementia, some studies suggest that its impairment might reflect a semantic decline (Rapcsak et al., 1989; Fromm et al., 1991; Patterson et al., 1994; O’Carroll et al.,1995; Storandt et al., 1995; Taylor et al., 1996; Strain et al., 1998; Glosser et al., 1999; Cockburn et al., 2000; Brambati et al., 2009). This hypothesis is in line with models of reading that consider the core influence of semantic processes on irregular word reading (Seidenberg et McClelland, 1989; Patterson et Hodges, 1992; Plaut et al., 1996; Coltheart, 2006; Woollams et al., 2007, Taylor et al., 2013; Taylor et al., 2015; Chapleau et al., 2017). Consistent with this idea, AD performances on reading and writing tasks that rely to a lesser extent on semantic processing (e.g., reading or writing of words with regular grapheme-phoneme mappings) appear to be qualitatively more similar to, than divergent from, normal performances, in contrast with tasks requiring semantic processing such as exception word reading (Glosser et al., 1999). This is further supported by a co-occurring and proportionally similar decline in semantic performances (as measured for instance by picture naming performance) and irregular word reading (Strain et al., 1998). Thus, it would appear that a core semantic memory deficit may be the underlying mechanism to impaired irregular word reading in AD dementia, in line with a large body of work suggesting that semantic memory impairments are an early and predominant symptom in MCI and AD dementia (Predovan et al., 2014; Montembeault et al,. 2017; Joubert et al., 2017; Montembeault et al., 2019; Joubert et al., 2021). Consistently with this hypothesis, the left anterior temporal lobe (ATL) region, involved in semantic processing (Shimotake et al., 2014), seems to play a critical role in irregular word reading tasks (Wilson et al., 2012; Hoffman et al. 2015; Joyal et al., 2017; Ueno et al., 2018), and shows atrophy in patients on the AD continuum (Joubert et al., 2010; Frings et al., 2011; Chapleau et al., 2016). Nonetheless, the hypothesis of a semantic deficit causing irregular word reading deficits in the AD continuum remains debated and more evidence is needed to draw solid conclusions regarding the underlying cognitive and neural mechanisms of irregular-word reading in these patients.

The aim of the present article is to assess over a large, well-characterized sample representative of the AD continuum, whether irregular word reading performances (a) significantly differ between diagnostic categories across this continuum and (b) are linked to general cognitive impairment / dementia severity. We hypothesize (1) that demographically-matched MCI and AD participants will perform significantly worse than controls on irregular word reading and (2) that irregular word reading will be correlated with general cognitive impairment / dementia severity. If these two hypotheses are supported by our results and that irregular-word reading performance is not maintained at different stages of AD, we will investigate three additional aims, namely whether the performance on irregular word reading is linked (c) to semantic neuropsychological tests; (d) to psycholinguistic variables associated with semantic processes (but not with psycholinguistic variables associated with phonological processes) and (e) to brain volumes in regions associated with semantic processing. These analyses will contribute to clarify the underlying cognitive and neural mechanisms of irregular word reading deficits. We hypothesize that (3) we will observe a stronger correlation between irregular word reading and tests of semantic processes (e.g. picture naming), as opposed to other tests (e.g., executive functions or episodic memory); (4) the accuracy of single items of the irregular words reading test will be associated with the lexicosemantic variables of the words (e.g., number of sense, semantic neighborhood, concreteness or age of acquisition) as opposed to phonological variables (e.g., number of phonemes, syllables or phonological neighborhood) and (5) finally, we should find neural correlates of semantics to be related to irregular word reading performance, namely the left ATL.

## Methods

The data used in the preparation of this article were obtained from the Alzheimer’s Disease Neuroimaging Initiative (ADNI) database (adni.loni.usc.edu). ADNI began in 2004 as a public–private partnership under the leadership of Dr. Michael W. Weiner. The primary goal of ADNI has been to detect AD dementia at the earliest possible stage (pre-dementia) and identify ways to track the disease progression. To that end, data from magnetic resonance imaging (MRI), positron emission tomography (PET), other biological markers as well as clinical and neuropsychological assessments have been collected to test if they can be combined to measure the progression of the various stages of the AD continuum. The initial five-year study (ADNI-1) was extended by two years in 2009 by a Grand Opportunities grant (ADNI-GO), and in 2011 and 2016 by further competitive renewals of the ADNI-1 grant (ADNI-2, and ADNI-3, respectively). For up-to-date information, see www.adni-info.org.

### 2.1 Participants

Participants over all ADNI studies (1, GO, 2 and 3) who had American National Adult Reading Test (AmNART) scores available at their baseline assessment were included in this study. All participants, aged between 54 and 91 years (inclusive), had completed a minimum of six years of education and did not have vascular dementia, depression, sensory disturbances, or other medical conditions that could interfere with the study. A study-partner who had frequent contact with the participant (an average of 10h per week or more) also accompanied them to visits and filled out questionnaires.

Participants were divided into five categories: healthy control (HC), subjective cognitive decline (SCD), early mild cognitive impairment (EMCI), late mild cognitive impairment (LMCI) and Alzheimer’s disease (AD) dementia.

The HC status was reserved for participants free of memory complaints, verified by a study partner, beyond what one would expect for age, as well as normal memory function documented by scoring above education adjusted cutoffs on the Logical Memory II subscale (LM II) delayed paragraph recall, from the Wechsler Memory Scaled - Revised (WMS-R). Scoring (a) ≥ 9 for 16 or more years of education; (b) ≥ 5 for 8–15 years of education; and (c) ≥ 3 for 0–7 years of education. Additionally, Mini-Mental State Examination (MMSE) score between 24 and 30 (inclusive), Clinical Dementia Rating (CDR) = 0, and without significant impairment in activities of daily living. There was no criterion regarding memory complaints.

Participants classified as SCD presented the same scores as HC participants on the WMS-R LM II, MMSE, CDR and presented no significant impairment in activities of daily living. Unlike their HC counterpart, SCD participants presented significant subjective memory concern as reported by subject, study partner, or clinician, as well as significant memory concern confirmed by Cognitive Change Index score ≥16.

Participants were classified as EMCI if they presented subjective memory concerns as reported by the subject, their study-partner or clinician, had abnormal memory function documented by scoring within the education adjusted ranges on the WMS-R LM II, scoring inclusively (a) 9–11 for 16 or more years of education; (b) 5–9 for 8–15 years of education; and (c) 3–6 for 0–7 years of education, an MMSE score between 24 and 30 (inclusive) and a CDR score = 0.5. Their general cognition and functional performance were sufficiently preserved so that a diagnosis of AD could not be made.

Participants were classified as LMCI if they presented subjective memory concerns as reported by the subject, their study-partner or clinician, had abnormal memory function documented by scoring within the education adjusted ranges on the WMS-R LM II, scoring (a) ≤ 8 for 16 or more years of education; (b) ≤ 4 for 8–15 years of education; and (c) ≤ 2 for 0–7 years of education, an MMSE score between 24 and 30 (inclusive) and a CDR score = 0.5. Their general cognition and functional performance were sufficiently preserved so that a diagnosis of AD could not be made.

Diagnosis of AD was made in participants with a memory complaint confirmed by a study partner (or reported only by the study-partner), with abnormal memory function documented by scoring within the education adjusted ranges on the WMS-R LM II, scoring (a) ≤ 8 for 16 or more years of education; (b) ≤ 4 for 8–15 years of education; and (c) ≤ 2 for 0–7 years of education, an MMSE score between 20 and 26 (inclusive), with a CDR score = 0.5 or 1, and who met the National Institute of Neurological and Communicative Disorders and Stroke and the Alzheimer’s Disease and Related Disorders Association criteria for probable AD.

In addition to ADNI general inclusion and group classification criteria, we applied for this study two additional specific criteria. The first one was to be native English speakers (excluded N = 33). The second criterion was consistency between total AmNART scores and single item-level data on this test, when available, in the ADNI database (excluded N = 52). Of the original 2097 and after all considerations, 2012 participants remained, of which 681 HC, 104 SCD, 290 EMCI, 589 LMCI and 348 AD. Demographics of this final sample are provided in the result section.

### 2.2 Procedure

#### 2.2.1 Cognitive assessments

##### AmNART.

To measure irregular word reading abilities in an American population, the AmNART (sometimes called ANART) was used. This test is an adaptation of the original British NART (Nelson, 1982) developed specifically for the American English population to estimate premorbid intelligence through irregular word reading (Grober and Sliwinski, 1991). The version used by ADNI comprises a list of 50 irregular words, with about half of them identical to the NART. These words are irregular words, also known as exception words, meaning that their actual pronunciation differs from what would be predicted based on the application of grapheme-to-phoneme mapping (e.g., pint, cellist). They are intended to be printed in order of increasing difficulty and are relatively short to avoid the possible adverse effect of stimulus complexity. Given no time limit, the subject is instructed to read aloud down the list of words, errors made in pronouncing each word is then recorded into an “error score”. Participants are allowed to self-correct but are not prompted to do so unless it was difficult to hear what was said and it is necessary to determine whether the pronunciation was correct or incorrect. If they hesitate on two different pronunciations, one correct and the other incorrect, they will be asked which one they think is best.

To assess the involvement of psycholinguistic variables on successful reading of irregular words, we extracted characteristics for each of the 50 AmNART irregular words using the English lexicon project (ELP; Balota et al., 2007) as well as the WordNet (Miller, 1995) data sets, prioritizing ELP, but using WordNet when data was not otherwise available. As control variables, we used (a) word length (number of letters), (b) objective lexical frequency, (c) orthographic neighborhood density and (d) summed bigram frequencies by position. The measure of lexical frequency was obtained from ELP and is the log 10 of number of times the word appears in the corpus +1. The measure of orthographic neighborhood density was the orthographic Levenshtein distance to the 20 closest neighbors in the lexicon (OLD20, Yarkoni et al., 2008). To put it simply, it is a measure of similarity and proximity to other words of the lexicon. Specifically, the OLD20 of a given word is computed as the mean of string edit distances from this word to its 20 closest orthographic neighbors in the lexicon. The edit distance used, Levenshtein distance (LD), corresponds to the number of operations (letter deletion, insertion, or substitution) needed to change a word into another word: for example, the LD from *smile* to *similes* is 2 (two insertions: I and S). Next is summed bigram frequencies by position, where bigram is defined as a sequence of two letters, it was obtained from ELP and is a measure of frequency of bigrams that is sensitive to positions within words by taking into account the letter positions where the bigram occurs. For example, the bigram frequency for DO in DOG counts DO bigrams only when they appear in the first two positions of a word in the corpus. As lexicosemantic variables, we used (a) age of acquisition, (b) concreteness, (c) number of senses and (d) semantic neighborhood density. The measure of age of acquisition was obtained from ELP, originally recorded by Kuperman and colleagues (2012) as the estimated age at which a word was learned, which has been shown to have larger effects in tasks involving semantic information (e.g., picture naming and lexical decision) as opposed to tasks where semantic information was less involved (e.g., reading aloud; Juhasz 2005; Elsherif et al., 2023). The measure of concreteness was obtained from ELP and is described by Brysbaert and colleagues (2014) as evaluating the degree to which the concept denoted by a word refers to a perceptible, relatable, entity. The measure for number of senses was obtained from WordNet and is described by Miller (1995) as the number contexts in which the word can be used to express the number of possible meanings it has. The measure for semantic neighborhood density was obtained from ELP and is described by Mirman and Magnuson (2008) as the number and/or proximity of neighboring representations, density referring to how tightly packed the words in the neighborhood are (Shaoul and Westbury, 2010). For phonological variables, we used (a) the number of syllables; (b) the number of phonemes (c) and phonological neighborhood. The measure of phonological neighborhood was obtained from ELP and is, similarly to the aforementioned OLD20, a measure of 20 phonological LD (PLD20).

##### Mini-Mental State Exam and Montreal Cognitive Assessment.

To measure general cognitive impairment/dementia severity, we used scores obtained by participants on the Mini Mental State Examination (MMSE; Folstein et al, 1975) and the Montreal Cognitive Assessment (MoCA; Nasreddine et al., 2005), two test that are routinely used to screen a wide range of cognitive functions and identify patients on the AD continuum, as well as to determine disease severity.

##### Boston naming test.

To measure lexicosemantic abilities, the Boston Naming Test (BNT; Kaplan et al., 1983) was used. It measures the ability to orally label (name) drawing of objects. Participants have 20 seconds to name what the drawing represents after being presented with the image. A semantic cue is given if the participant fails to recognize the picture (e.g., answering bench instead of tree) or if they state that they do not know what the picture represents. The semantic cue is either a short explanation about the item (e.g., for a mask: “it’s part of a carnival fantasy”) or a superordinate category (e.g., for a beaver: “it’s a kind of animal”). The test presents objects in order of frequency, from most to least common and is discontinued after 6 consecutive failures. ADNI only administers odd numbered items on the standard 60 item BNT, this gives us a maximum score of 30.

##### Trail making Part-B.

To measure executive functioning, the trail making test was used. More specifically, we used scores obtained on part-B of the test, which depends on visuomotor, perceptual-scanning skills and requires considerable cognitive flexibility in shifting from number to letter sets under time pressure (Partington and Leiter, 1949). 25 circles are presented to the participant which contains numbers 1 through 13 and letters A through L, the circles are scrambled across the given medium, the participant must connect the circles while alternating between numbers and letters in ascending order (e.g., A to 1; 1 to B; B to 2; 2 to C), they have up to 300 seconds to complete the test, their time to complete it (in seconds) is recorded as their score.

##### Rey Auditory Verbal Learning Test (30-minute delay).

To measure episodic memory, we used the Rey Auditory Verbal Learning Test (AVLT; Rey, 1964). Over five learning trials, participants are read a list of 15 words (list A), they are asked to recall them immediately with no regards for order. After the fifth learning trial, the same task is done using an interfering list (B). Immediately and 30 minutes after administration of list B, list A is recalled, this time without first being read. Scores from the 30-minute delay test were used as our measure of episodic memory.

#### 2.2.2 Neuroimaging

All participants received T1-weighted (T1w) MRIs (see http://adni.loni.usc.edu/methods/mri-tool/mri-analysis/ for the detailed MRI acquisition protocols). T1w scans for each participant were pre-processed through our standard pipeline including denoising (Coupe et al., 2008), intensity inhomogeneity correction (Sled et al., 1998) and intensity normalization into range [0–100]. The pre-processed images were then both linearly (9 parameters: 3 translation, 3 rotation, and 3 scaling; Dadar et al., 2018) and nonlinearly (Avants et al., 2008) registered to a population appropriate average template generated based on 150 ADNI participants. The quality of all the image processing steps, including the linear and nonlinear registrations was visually verified by an experienced rater (MD). Deformation-based morphometry (DBM) was performed to measure the local anatomical differences in the brains of the participants by estimating the Jacobian determinant of the inverse of the estimated nonlinear deformation field as a proxy of atrophy (Dadar et al., 2020). DBM values reflect the relative volume of the voxel with respect to the template; i.e. a value of 1 indicates similar volume to the same region in the template, values lower than one indicate volumes smaller than the corresponding region in the template, while values higher than one indicate volumes that are larger than the corresponding region in the template. Therefore, lower DBM values can be interpreted as reduction in the structure volume, i.e., regional atrophy. Voxel-wise DBM maps were used to assess the relationship between brain atrophy and AmNART scores at a voxel level. In addition, mean DBM values within a region of interest (ROI) including the left anterior temporal lobe were used to assess the relationship between atrophy in the left anterior temporal lobe and AmNART scores.

### 2.3 Statistical analyses

#### 2.3.1 Behavioral analyses

To describe the sample, Pearson’s chi-square test was used to assess sex differences. One-way analysis of variance (ANOVA) and Tukey post-hoc testing were used for all other variables.

To test the hypotheses that AmNART scores are dementia insensitive and semantic-related we modeled a number of multiple linear regression that predicts AmNART total error score based on (1) diagnostic category, extracting an ANOVA table to test for the factor as a whole, (2) tests of severity (MMSE, MoCA) and (3) neuropsychological tests (BNT, TRAIL-B, AVLT), controlling for sex, age, and education, as well as severity as measured by the MMSE in when assessing relation to neuropsychological tests. The MMSE was favored to control for severity as results on the MoCA were not available for the whole sample.

Epsilon square was used as a measure for effect size (ε^2^; Okada, 2013). Stein’s formula was used to calculate adjusted *R*^2^ (Stevens, 2002).

To assess the involvement of each psycholinguistic variable extracted from the AmNART (as presented in 2.2.1) on irregular word reading, we analyzed single-item accuracy with a generalized logistic mixed-effects model using the Ime4 package (Bates et al., 2015). This analysis was conducted on a subsample of participants who had single item-level AmNART data available, as opposed to only having total AmNART score available (195 HC, 323 LMCI, 156 AD). Single-item accuracy was predicted by length, lexical frequency, orthographic neighborhood, bigram frequencies by position, age of acquisition, concreteness, number of senses, semantic neighborhood density, number of syllables, number of phonemes and phonological neighborhood as fixed effects, with by-item and by-subject random intercepts as random effects. |z| values beyond 1.96 were deemed as significant (Baayen, 2008). Bigram frequencies by position and number of senses were logarithmically transformed to normalize these variables. 20 words with missing values in age of acquisition, objective lexical frequency, concreteness, and/or phonological neighborhood had to be excluded from this analysis. The remaining 30 words were ache, aisle, algae, asthma, blatant, bouquet, cellist, chord, courteous, debt, deny, depot, epitome, façade, gauge, heir, hiatus, hyperbole, naïve, nausea, papyrus, pint, placebo, scion, sieve, simile, subtle, superfluous, thyme and zealot.

#### 2.3.2 Neuroimaging analyses

Similar linear regression models were used to assess the relationship between AmNART scores and voxel-wise DBM values in the subset of the participants that had MRI information available (N = 1863), controlling for age, sex, and level of education. A second set of models were also run with diagnostic category as an additional covariate. Voxel-wise results were corrected for multiple comparisons using False Discovery Rate (FDR) controlling technique, with a significance threshold of 0.05.

Second, we conducted a ROI-based analysis to test the specific hypothesis of a relationship between the volume in the left ATL and irregular word reading on a subsample of participants who had neuroimaging data available (N = 1863). To do so, we modeled a multiple linear regression that predicts AmNART total error score based on the DBM in the left ATL, controlling for sex, age, education, with and without including diagnostic category as a covariate in the models, similar to the voxel level analyses. The ATL ROI was selected from a previous study (Borghesani et al., 2020, Geraudie et al., 2023).

All statistical analyses were performed using R Statistical Software (version 4.2.1; R Foundation for Statistical Computing, Vienna, Austria).

## Results

Demographic characteristics of the 2012 participants are shown in table 1. Groups differed with regards to sex *χ*^2^ (4) = 44.94, *p<* 0.001, age *F*(4, 2007) = 12.15, *p<* 0.001, ε^2^ = 0.02 as well as education *F*(4, 2007) = 12.57, *p<* 0.001, ε^2^ = 0.02. All following analyses were therefore controlled for sex, age and education. Neuropsychological and language evaluations broadly revealed the expected patterns of impairment across the AD continuum. First, measures of severity worsened along the continuum of disease progression stages. Second, episodic memory deficits are predominant, but cognitive decline gradually extends to other cognitive domains.

### 3.1 Irregular word reading across the AD continuum

**Figure 1:**
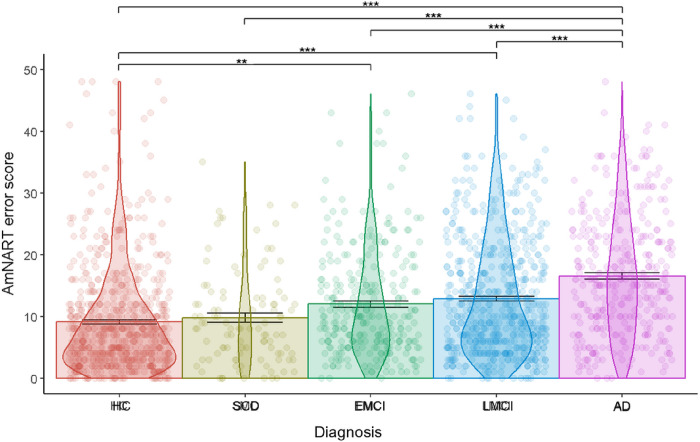
Relation between AmNART error score and diagnostic category

When controlling for sex, age, education, AmNART total error score significantly differed between diagnoses (*F*[4,2004] = 52.20 *p<* .001, partial ε^2^ = .09, Fig. 1). Overall, patient groups with more advanced disease progression on the AD continuum made more errors on irregular word reading. Specifically, as seen in Fig. 1, AD dementia participants showed significantly lower performance compared to all other groups. In addition, HC scores also differed significantly from that of EMCI and LMCI. Means and standard deviations of AmNART total scores as well as significant differences are presented in Table 2 (more detailed *T* ratios, *p* values and effect sizes for each contrast are presented in supplementary table 1).

Of note, we observed the presence of 18 outlier participants (AmNART total error score deviated by ± 3.29 z-scores in comparison to the average and standard-deviation of their respective diagnostic group), more precisely 12 HC, 3 EMCI and 3 LMCI. However, excluding these participants did not impact any of the results of the analyses.

### 3.2 Association between irregular word reading and general cognitive impairment / severity

**Figure 2:**
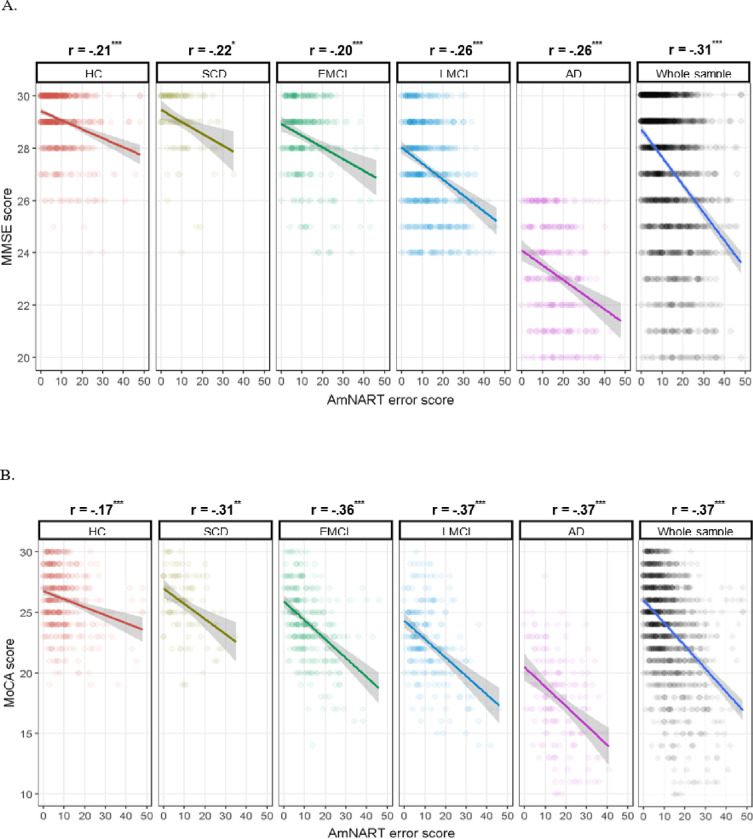
A. Relation between MMSE and AmNART error score relative to diagnostic category B. Relation between MoCA and AmNART error score relative to diagnostic category *Note.* *: *p* < .05, **: *p* < .01, ***: *p* < .001.

Whole sample and group-specific partial correlations between AmNART and measures of disease severity/global cognition (MoCA and MMSE) are presented in Fig. 2. Both measures of severity were significantly correlated with total AmNART scores, in all diagnostic groups as well as across the whole sample, further supporting a strong link between AD disease progression and impaired irregular word reading.

### 3.3 Association between irregular word reading and lexicosemantic, executive functioning and episodic memory performances

**Figure 3:**
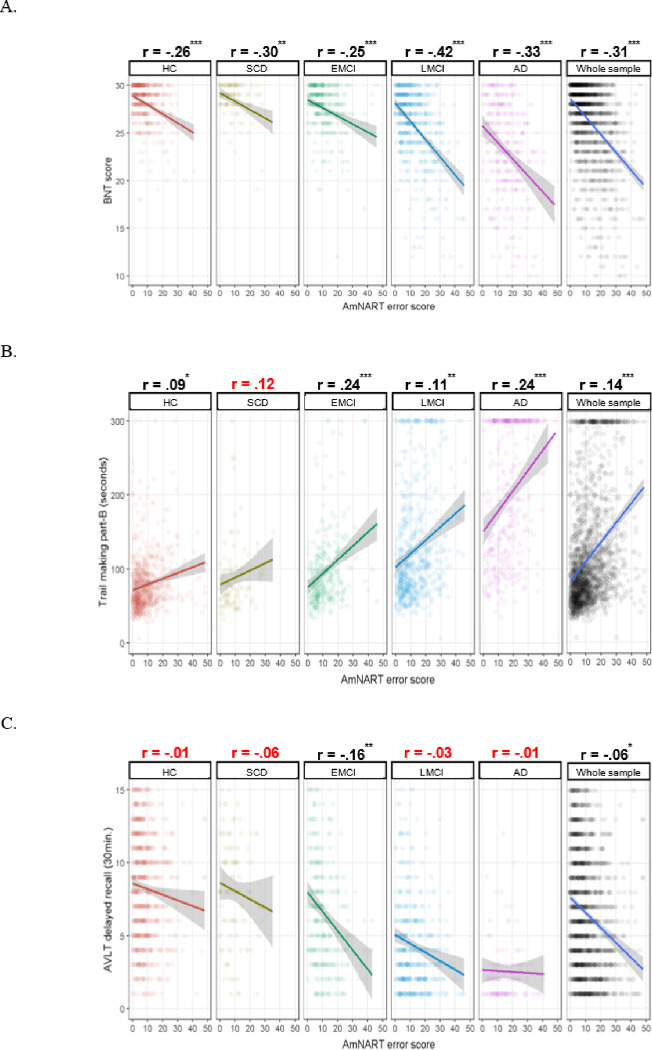
A. Relation between Boston Naming Test and AmNART error score relative to diagnostic category B. Relation between Trail making part-B and AmNART error score relative to diagnostic category C. Relation between AVLT delayed recall and AmNART error score relative to diagnostic category *Note.* *: *p* < .05, **: *p* < .01, ***: *p* < .001.

Whole-sample and group-specific partial correlations between AmNART and the chosen neuropsychological tests (BNT, Trail making part-B and AVLT delayed recall) are presented in Fig. 3. Total AmNART irregular word reading scores were significantly and moderately correlated with BNT scores (measuring picture naming or lexicosemantic abilities), weakly but significantly correlated with the Trail making part-B (measuring executive functioning), and poorly correlated with the AVLT delayed recall (measuring episodic memory), being only significant in the EMCI group (p < .001) and across the whole sample (p < .05).

The model created to distinguish between the involvement of lexicosemantic, executive and memory functions in irregular word reading is presented in Table 2. Consistently with the correlational analyses, we observed that the BNT provides a strong contribution to the model (standardized *β*= −0.31, *p<* .001), the trail making provides a weak but significant contribution (standardized *β* = −0.06, *p<* .001 ) and the AVLT delayed recall does not provide a significant contribution (p= .887).

### 3.4 Association between irregular words and psycholinguistic variables (lexicosemantic and phonological)

To better understand the relationships between AmNART irregular words and correct reading, we first selected a subsample for whom single item-level AmNART data was available (195 HC, 323 LMCI, 156 AD). The model used to predict irregular word item success based on their psycholinguistic variables is presented in Table 3. While none of the phonological variables had a significant effect on irregular word reading accuracy, there was a significant effect of age of acquisition *(β* = −0.42, *z* = −5.62).

### 3.5 Link between irregular word reading and brain volumes

**Figure 4:**
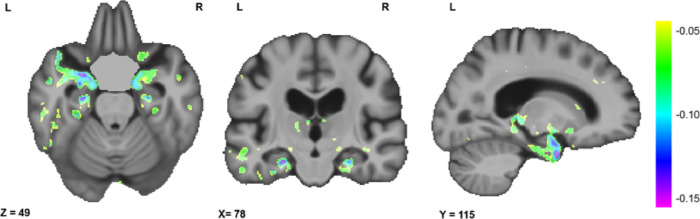
Relation between voxel-wise DBM maps and AmNART error score Axial, coronal and sagittal slices showing the t-statistic maps reflecting the significant patterns of brain volume changes in the sample. Colour gradient indicates shrinkage of the tissue (i.e., atrophy). X, Y and Z values indicate MNI coordinates for the displayed slice.

Figure 4 shows the results of the significant associations between voxel-wise DBM maps and AmNART scores, including age, sex, and education level as covariates, after correction for multiple comparisons (FDR). At a voxel-wise whole brain level, we observed significant correlations with bilateral medial temporal lobe regions, including the hippocampi, as well as with the ATL, the inferior and middle temporal gyrus, and the fusiform gyrus, predominantly in the left hemisphere. However, no voxels survived FDR correction after including the diagnostic group as covariate. At the ROI level, ATL DBM values were significantly associated with AmNART scores when including age, sex, and education as covariates (standardized *β*= −0.11, *p<* .001). Furthermore, this association remained significant after including diagnostic group as an additional covariate (standardized *β* = −0.05, *p<* .05). The model used to predict AmNART error score based on brain volumes in the ATL is presented in Table 4.

Relation between voxel-wise DBM maps and AmNART error score

## Discussion

The present study aimed to assess, over a large and well-characterized sample of participants on the AD continuum, whether irregular word reading performance is an accurate indicator of premorbid intelligence, or a marker of general cognitive and semantic deficits. Results showed that EMCI, LMCI and AD patients make significantly more errors in reading irregular words compared to HC, and that AD patients also make significantly more errors than all other groups. Across the whole AD continuum, as well as within each diagnostic group, irregular word reading abilities were further significantly correlated to measures of general cognitive impairment / dementia severity. This suggests that irregular word reading performances decline throughout the AD continuum, and that even at a finer grain beyond diagnostic categories, a strong link exists between dementia severity and irregular word reading difficulties. Premorbid IQ estimates based on this measure should therefore not be considered accurate in patients with MCI or AD. Furthermore, results indicated significant moderate association between irregular word reading and neuropsychological tests of lexicosemantics, as opposed to weak association to executive function and no association to episodic memory. At the item-level, none of the phonological variables had significant effect on irregular word reading accuracy whilst age of acquisition, a semantic variable, provided a significant contribution. Finally, the whole-brain neuroimaging analysis pointed to the hippocampal and left ATL volume loss as the main contributors to decreased irregular word reading performances. These results are consistent with the theory of irregular word reading impairments as an indicator of disease severity and semantic decline, as opposed to an indicator of premorbid IQ, and pave the way for further investigation on the matter.

Consistent with our first hypothesis, MCI and AD participants performed significantly worse than controls in reading of irregular words, controlling for sex, age and education. EMCI, LMCI and AD participants correctly read an average of 2.9,3.8 and 7.4 fewer words, respectively, than HC. These measures are comparable to that of Weinborn and colleagues (2018) who, when using the Wechsler Test of Adult Reading (WTAR, another 50 irregular word test) found that MCI and AD participants read on average 3.0 and 7.4 fewer words, respectively, than HC. Consistent with hypothesis 2, results indicate that irregular word reading is correlated with general cognitive impairment / dementia severity. This relationship was similar in controls as it was throughout the different diagnostic categories, although and expectedly, that relationship becomes stronger as we advance throughout the AD continuum, when larger variations in impairment appear. Taken together, these two sets of results indicate that irregular word reading performances decline throughout the AD continuum and that estimates of premorbid IQ based on this measure should not be considered accurate in patients with MCI and AD. Indeed, the assessment of premorbid IQ with the AmNART in participants on the AD continuum violates the criteria set by Taylor and colleagues in 1996, that is to say that an accurate estimate of premorbid IQ should (a) not significantly change as disease progresses in severity and (b) not differ significantly from those of demographically matched control subjects. This has major clinical implications: given that irregular word reading estimates of premorbid IQ are inaccurate in this population, clinicians and researchers could be led to underestimate cognitive changes in people with memory complaints, be more likely to underdiagnose AD continuum conditions or underestimate disease progression in those already diagnosed with one of these conditions. Therefore, it seems preferable for clinicians to rely on comparisons to demographically-adjusted norms of cognitive performance to establish cognitive decline, as well as on repeated measures overtime.

Consistent with hypothesis 3, lexicosemantic abilities were the second-best predictor of irregular word reading performances, just after education but largely above dementia severity and other cognitive functions (executive functions and episodic memory). The importance of lexicosemantic abilities in irregular-word reading was further in line with hypothesis 4, as AmNART item success rate was significantly predicted by the age of acquisition of irregular words, which has been associated with semantic representations (Juhasz 2005; Elsherif et al., 2023). This is consistent with the fact that NART-like tests are intended to bypass phonemic decoding by relying more heavily on a person’s knowledge of exceptional spelling associated with irregular words. Overall, this set of results highlights the strong association between irregular word reading and semantic abilities, as suggested by Strain and colleagues in 1998 and consistent with the idea of semantic abilities’ core influence on irregular word reading performances, particularly but not limited to the AD continuum population. These results are consistent with models of reading that would consider the core influence of semantic abilities on correct reading aloud of irregular words, as emphasized by Taylor and colleagues in their 2015 review. Although not all semantic psycholinguistic variables significantly predicted correct reading, the significant involvement of age of acquisition is consistent with the idea that words acquired at a younger age and used more frequently within the population might be more strongly stored in semantic memory, enhancing the likelihood of successful reading. What these results also show is that executive functioning, episodic memory and phonology do not seem to be as crucial in irregular word reading performance.

Beyond the implication of lexicosemantic abilities as an underlying cognitive mechanism of irregular word reading, we were also interested in looking at the underlying neural mechanisms. Results of our neuroimaging analyses were in line with hypothesis 5. The whole-brain analysis suggested that bilateral hippocampi volumes, as well as with the ATL, the inferior and middle temporal gyrus, and the fusiform gyrus, predominantly in the left hemisphere, were strongly related to AmNART performance, The significant correlation with the ATL, even when controlling for diagnostic groups, was further confirmed in the ROI-based analysis. Atrophy of the hippocampus is a marker of AD disease severity. The hippocampus is known to be one of the key brain structures affected in AD dementia (Rao et al., 2022), being the primary site of accumulation of beta-amyloid proteins and phosphorylated tau (Chu, 2012) as well as presenting markedly more degeneration than in other neurodegenerative diseases affecting hippocampal volumes (Jaroudi et al., 2017; Rao et al., 2022). Since we found that AmNART scores are also highly correlated with disease severity, it is therefore unsurprising that AmNART scores and hippocampal volumes would be strongly associated. In addition to the well-documented involvement of the hippocampus in episodic memory, research also shows it could well be involved in semantic memory processes (Binder at al., 2009; Duff and Brown-Schmidt, 2012; Piai et al., 2016; Chapleau et al., 2019).

The significant involvement of the ATL in irregular word reading performances in ROI-based analyses is consistent with previous results supporting the involvement of the ATL in irregular word reading (Wilson et al., 2012; Hoffman et al. 2015; Joyal et al., 2017; Ueno et al., 2018). These observations in AD patients are not dissimilar to ATL atrophy in semantic dementia, also accompanied with irregular word reading deficits (Woollmans et al., 2007). However, single word reading tasks like the AmNART have been hypothesized to not be demanding enough on the ATL (Taylor et al., 2013) which could explain the small effect size. Taken together, these results are consistent with the theory of irregular word reading impairments as an indicator of general cognitive decline and semantic decline, as opposed to an indicator of premorbid intelligence.

While the current study fulfills many gaps in the literature (large sample size, well-characterized participants at four different stages on the AD continuum, investigation of underlying cognitive and neural mechanisms of irregular word reading), these results also need to be considered within the context of several limitations. Firstly, the cross-sectional design of the study does not confirm that irregular word reading declines with time in participants on the AD continuum, as a longitudinal design would. Secondly, the ADNI cohort is not population-based and underrepresents ethnoculturally diverse populations, its participants are also highly educated and have fewer comorbidities compared to other cohorts (Birkenbihl et al., 2020). ADNI results must be interpreted with the caveat that they may have limited external validity for more diverse populations. Generalizing to other populations is further complicated by differences in how irregular words are experienced in other languages with more transparent spelling-to-sound correspondences. Italian for example, is more transparent than the more opaque English and is characterized by regular spelling to sound correspondence (Colombo et al., 2000. The same can be said for languages that incorporate phonograms or ideograms (e.g., Chinese, Japanese, Korean or Vietnamese) which could be more context-dependent or invoke greater imageability in reading. Third, item-level analyses were conducted on a subsample of participants for which item-level AmNART data (as opposed to only total AmNART score) was available (195 HC, 323 LMCI, 156 AD). Additionally, 20 words with missing values in age of acquisition, objective lexical frequency, concreteness, and/or phonological neighborhood had to be excluded from item-level analyses. Fourth, the use of the BNT as unique semantic test has certain limits, as picture naming involves distinct cognitive processes that are not limited to semantics. Involved are visual analysis of the picture, recognition of the stimulus as familiar, activation of the semantic representation of the object via the semantic system, a lexical-semantic process which directs selection and retrieval of semantic information in a task appropriate way, modality-independent lexical access to the phonological word form of the object, that is to say the speech sounds used in the word; and the motor programming and articulation required for saying the word (DeLeon et al., 2007; Harry and Crowe, 2014). This is important as reading models see the involvement of both lexical and semantic processing in correct reading of irregular words (Taylor et al., 2013), these results should therefore not be interpreted as an involvement of semantics alone.

## Conclusions

Measuring cognitive decline can be particularly challenging for clinicians when considering that diseases as insidious as AD dementia may be involved. Cognitive decline will more often than not have to be estimated post-hoc, blind to an individual’s objective baseline performances. The first assessment, where only one time point is available, could prove critical to any intervention against the disease and its progression. Currently, clinicians have to rely on subjective complaints, demographically-adjusted norms of cognitive performance and repeated measures. The results of this study lend support to the idea that irregular word reading tests do not provide an accurate estimate of premorbid IQ in the MCI-AD populations as it appears irregular word reading performances significantly declines in this population and are related to semantic impairments correlated to hippocampal and ATL volume loss. Relying on these estimates could lead clinicians to underestimate cognitive decline in people with those conditions. Premorbid estimates should rely on more crystalized forms of intelligence that are uncorrelated to disease severity as evidenced by longitudinal studies in clinically diverse populations.

## Figures and Tables

**Table 1:** Demographics, neuropsychological and language data for all groups

	HC (n=681)	SCD (n=104)	EMCI (n=290)	LMCI (n=589)	AD (n=348)	P value
Sex, M/F	304/377	42/62	161/129	362/227	196/152	**<. 001**
Age, years	72.8 ± 6.3^c,e^	72.2 ± 5.6^e^	71.3 ± 7.6^a,d,e^	73.7 ± 7.6 ^c^	74.9 ± 7.9^a,b,c^	**<. 001**
	55–90	60–90	55–89	54–91	55–91	
Education, years	16.5 ± 2.6^c,d,e^	16.8 ± 2.5^e^	16.0 ± 2.7^a,e^	16.0 ± 2.8^a,e^	15.3 ± 2.9^a,b,c,d^	**<. 001**
**Global cognition / Severity**						
CDR	0.0 ± 0.0^c,d,e^	0.0 ± 0.0^c,d,e^	0.5 ± 0.0^a,b,e^	0.5 ± 0.0^a,b,e^	0.8 ± 0.3^a,b,c,d^	**<. 001**
MoCA	26.2 ± 2.4 (458)^c,d,e^	25.7 ± 2.6 (102)^c,d,e^	24.1 ± 3.0 (287)^a,b,d,e^	22.5 ± 3.2 (234)^a,b,c,e^	17.2 ± 4.5 (173)^a,b,c,d^	**<. 001**
MMSE	29.1 ± 1.1^c,d,e^	29.0 ± 1.2^c,d,e^	28.4 ± 1.5^a,b,d,e^	27.2 ± 1.8^a,b,c,e^	23.2 ± 2.0^a,b,c,d^	**<. 001**
**Episodic memory**						
AVLTdeyaled recall (30min.)	8.2 ± 3.7 (649)^c,d,e^	8.1 ± 3.6 (99)^c,d,e^	6.5 ± 3.8 (259)^a,b,d,e^	4.3 ± 3.2 (392)^a,b,c,e^	2.5 ± 2.2 (96)^a,b,c,d^	**<. 001**
Logical memory, delayed recall	13.3 ± 3.3^c,d,e^	13.2 ± 3.2^c,d,e^	8.9 ± 1.6^a,b,d,e^	3.9 ± 2.6^a,b,c,e^	1.3 ± 1.8^a,b,c,d^	**<. 001**
**Language**						
Object naming (BNT, 30-item)	28.1 ± 2.1 (399)^d,e^	28.4 ± 1.9^d,e^	27.5 ± 2.6 (289)^d,e^	25.7 ± 4.0 (509)^a,b,c,e^	22.35 ± 5.9 (305)^a,b,c,d^	**<. 001**
Semantic fluency (animals)	21.2 ± 5.6^c,d,e^	20.0 ± 5.3^d,e^	18.8 ± 5.1^a,d,e^	16.5 ± 5.0^a,b,c,e^	12.3 ± 5.0^a,b,c,d^	**<. 001**
Phonemic fluency (f-words)	14.8 ± 4.7 (461)^c,d,e^	14.1 ± 4.3 (103)^e^	13.7 ± 4.8^a,e^	13.7 ± 4.4 (237)^a,e^	10.9 ± 4.5 (179)^a,b,c,d^	**<. 001**
**Executive functioning**						
Trail making Part-A (seconds)	32.9 ± 11.3^d,e^	34.1 ± 36.8^d,e^	36.8 ± 14.7^d,e^	43.6 ± 21.5^a,b,c,e^	63.6 ± 35.2 (344)^a,b,c,d^	**<. 001**
Trail making Part-B (seconds)	78.3 ± 37.4 (678)^c,d,e^	88.3 ± 45.7^d,e^	97.9 ± 50.7 (286)^a,d,e^	125.9 ± 71.8 (581)^a,b,c,e^	196.0 ± 87.0 (315)^a,b,c,d^	**<. 001**
**Visuospatial functioning**						
Copy of a clock	4.7 ± 0.6 (680)^d,e^	4.7 ± 0.6^d,e^	4.6 ± 0.7^d,e^	4.2 ± 1.0^a,b,c,e^	3.4 ± 1.4 (347)^a,b,c,d^	**<. 001**
**Irregular word reading**						
AmNART (error score)	9.1 ± 8.4^c,d,e^	9.8 ± 7.7^d,e^	12.0 ± 8.7^a,e^	12.9 ± 9.3^a,b,e^	16.5 ± 9.6^a,b,c,d^	**<. 001**
	0–48	0–35	0–46	0–46	0–48	

*Note*. Groups means +/− standard deviation results of demographic, cognitive and language characteristics. Numbers in brackets indicate numbers of participants with the score when less than total. Abbreviations: M: male, F: female, CDR: Clinical Dementia Rating Scale, MoCA: Montreal Cognitive Assessment, MMSE: Mini-Mental State Exam, AVLT: Auditory Verbal Learning Test (which includes a 30-minute delayed recall), BNT: Boston Naming Test, AmNART: American National Adult Reading Test.

a : differed significantly from HC,

b : differed significantly from SCD,

c : differed significantly from EMCI,

d : differed significantly from LMCI,

e : differed significantly from AD.

**Table 2:** Multiple regression predicting AmNART total error score using neuropsychological tests results

	adjusted R^2^	B	SE B	β	P
Model	0.39				< .001
Constant		76.77	4.39		< .001
**Control variables**					
Sex		−1.15	0.08	−0.13*	< .001
Age		−0.10	0.03	−0.09*	< .001
Education		−2.30	0.43	−0.38*	< .001
Severity: MMSE		−0.64	0.12	−0.15*	< .001
**Neuropsychological tests**					
Lexicosemantic: Boston Naming Test		−0.79	0.07	−0.31*	< .001
Executive function: Trail making part-B		0.01	0.00	0.06*	< .05
Episodic memory: AVLTdeyaled recall (30min.)		0.01	0.06		.887

**Table 3:** Generalized logistic mixed-effects model predicting irregular word successful reading using psycholinguistic variables

	B	SE B	z
Control variables			
Length (number of letters)	0.39	0.25	1.54
Frequency (log10)	0.84	0.45	1.89
Orthographic neighborhood (OLD20)	−0.19	0.50	−0.39
Bigram frequencies by position (log)	−0.88	0.37	−2.40
**Lexicosemantic variables**			
Age of Acquisition	−0.42	0.07	−5.62
Concreteness	−0.07	0.16	−0.46
Number of senses (log)	0.17	0.25	0.69
Semantic neighborhood density	2.40	1.41	1.70
**Phonological variables**			
Number of syllables	−0.52	0.30	−1.71
Number of phonemes	0.48	0.32	1.49
Phonological neighborhood (PLD20)	−0.55	0.44	−1.24

*Note*. z values beyond 1.96 in absolute values are deemed as significant (Baayen, 2008).

**Table 4:** Multiple regression predicting AmNART total error score using anterior temporal lobe volume

	adjusted R^2^	B	SE B	β	P
Model	0.27				< .001
Constant		36.23	3.00		< .001
**Control variables**					
Sex		−2.24	0.36	−0.13*	< .001
Age		−0.01	0.02	−0.01*	0.696
Education		−1.41	0.07	−0.44*	< .001
Diagnostic category		1.23	0.13	0.21*	< .001
**Region of interest**					
Anterior temporal lobe		−3.23	3.00	−0.05*	< .05

## Data Availability

Data analysed in this study were acquired from the ADNI database (http://adni.loni.usc.edu). All ADNI data are shared without embargo through the Laboratory of Neuro Imaging and Data Archive. Interested scientists may obtain access to ADNI imaging, clinical, genomic, and biomarker data for the purposes of scientific investigation, teaching, or planning clinical research studies. Access is contingent on adherence to the ADNI Data Use Agreement and the publications’ policies. For further information, please refer to the ADNI website (http://adni.loni.usc.edu/data-samples/access-data/).

## List Of Abbreviations

AD: Alzheimer’s disease
AD NI: Alzheimer’s Disease Neuroimaging Initiative
Am N ART: American National Adult Reading Test
ANOVA: Analysis of variance
ATL: Anterior temporal lobe
AVLT: (Rey) Auditory Verbal Learning Test
BNT: Boston Naming Test
CDR: Clinical Dementia Rating
DBM: Deformation-based morphometry
ELP: English lexicon project
EMCI: Early mild cognitive impairment
FDR: False Discovery Rate
HC: Healthy controls
IQ: Intellectual quotient
LD: Levenshtein distance
LM II: Logical Memory II subscale
LMCI: Late mild cognitive impairment
MCI: Mild cognitive impairment
MMSE: Mini-Mental State Examination
MoCA: Montreal Cognitive Assessment
MRI: Magnetic resonance imaging
NART: National Adult Reading Test
OLD20: Orthographic Levenshtein distance to the 20 closest neighbors
PET: Positron emission tomography
PLD20: Phonologic Levenshtein distance to the 20 closest neighbors
ROI: Region of interest
SCD: Subjective cognitive decline
Tlw: T1-weighted
TRAIL-B: Trail making test part B
AD: Alzheimer’s disease
WMS-R: Wechsler Memory Scaled - Revised
